# ^•^BMPO-OOH Spin-Adduct as a Model for Study of Decomposition of Organic Hydroperoxides and the Effects of Sulfide/Selenite Derivatives. An EPR Spin-Trapping Approach

**DOI:** 10.3390/antiox9100918

**Published:** 2020-09-26

**Authors:** Anton Misak, Vlasta Brezova, Marian Grman, Lenka Tomasova, Miroslav Chovanec, Karol Ondrias

**Affiliations:** 1 Department of Molecular Physiology, Institute of Clinical and Translational Research, Biomedical Research Center, Slovak Academy of Sciences, Dúbravská cesta 9, 84505 Bratislava, Slovakia; anton.misak@savba.sk (A.M.); marian.grman@savba.sk (M.G.); lenka.tomasova@savba.sk (L.T.); 2 Faculty of Chemical and Food Technology, Institute of Physical Chemistry and Chemical Physics, Slovak University of Technology in Bratislava, Radlinského 9, 81237 Bratislava, Slovakia; vlasta.brezova@stuba.sk; 3 Department of Genetics, Cancer Research Institute, Biomedical Research Center, Slovak Academy of Sciences, Dúbravská cesta 9, 84505 Bratislava, Slovakia; miroslav.chovanec@savba.sk

**Keywords:** hydroperoxides, antioxidants, EPR spectra simulation, ^•^BMPO-OOH spin-adduct, superoxide, radical, hydrogen sulfide, polysulfides, selenite, DNA

## Abstract

Lipid hydroperoxides play an important role in various pathophysiological processes. Therefore, a simple model for organic hydroperoxides could be helpful to monitor the biologic effects of endogenous and exogenous compounds. The electron paramagnetic resonance (EPR) spin-trapping technique is a useful method to study superoxide (O_2_^•−^) and hydroxyl radicals. The aim of our work was to use EPR with the spin trap 5-*tert*-butoxycarbonyl-5-methyl-1-pyrroline-*N*-oxide (BMPO), which, by trapping O_2_^•−^ produces relatively stable ^•^BMPO-OOH spin-adduct, a valuable model for organic hydroperoxides. We used this experimental setup to investigate the effects of selected sulfur/selenium compounds on ^•^BMPO-OOH and to evaluate the antioxidant potential of these compounds. Second, using the simulation of time-dependent individual BMPO adducts in the experimental EPR spectra, the ratio of ^•^BMPO-OH/^•^BMPO-OOH—which is proportional to the transformation/decomposition of ^•^BMPO-OOH—was evaluated. The order of potency of the studied compounds to alter ^•^BMPO-OOH concentration estimated from the time-dependent ^•^BMPO-OH/^•^BMPO-OOH ratio was as follows: Na_2_S_4_ > Na_2_S_4_/SeO_3_^2−^ > H_2_S/SeO_3_^2−^ > Na_2_S_2_ ~Na_2_S_2_/SeO_3_^2−^ ~H_2_S > SeO_3_^2−^ ~SeO_4_^2−^ ~control. In conclusion, the presented approach of the EPR measurement of the time-dependent ratio of ^•^BMPO-OH/^•^BMPO-OOH could be useful to study the impact of compounds to influence the transformation of ^•^BMPO-OOH.

## 1. Introduction

Exogenously added and endogenously produced hydrogen sulfide (H_2_S) and polysulfides affect many physiological and pathologic processes [1,2,3,4]. They modulate oxidative stress by reacting with reactive oxygen and nitrogen species [1,5,6,7]. Selenium (Se) is an essential trace element for humans, with multiple and complex effects on health, having antioxidant properties due to its presence in 25 selenoproteins in the form of selenocysteine amino acid [8]. Se compounds and H_2_S are present in living organisms and either alone or in combination interact with reactive oxygen species (ROS) [1,9,10,11,12]. In our previous work, we found that products of the sulfide/selenite (H_2_S/SeO_3_^2−^) interaction scavenge superoxide-derived radicals, cleave plasmid DNA (pDNA) and modulate tonus of isolated rat aorta and blood pressure [13]. However in this work, using the procedure of addition of KO_2_ as a source of (O_2_^•−^) into the mixture of the compounds (H_2_S, SeO_3_^2−^) with the spin-trapping agent 5-*tert*-butoxycarbonyl-5-methyl-1-pyrroline *N*-oxide (BMPO) it was not clear which components of the electron paramagnetic resonance (EPR) spectra resulted from the compounds/O_2_^•−^ or compounds/^•^BMPO-OOH interactions. To solve this issue, a new experimental strategy for the interaction of the compounds with ^•^BMPO-OOH only is herein presented.

Lipid peroxidation is a process under which oxidants such as free radicals attack lipids containing carbon-carbon double bond(s), leading to the radical chain reactions. It has an important role in cell biology and in various pathophysiological processes in which lipid hydroperoxides display a crucial function [14]. Importantly, many pathophysiological states can be regulated by the modulation of lipid peroxidation induced by exogenous compounds. Therefore, simple models of organic hydroperoxide could be useful for studying the effects of various compounds. EPR spin-trapping technique using cyclic nitrone BMPO is a reliable method to study O_2_^•−^ and hydroxyl radicals (HO^•^) [15,16,17].

**Scheme 1:**
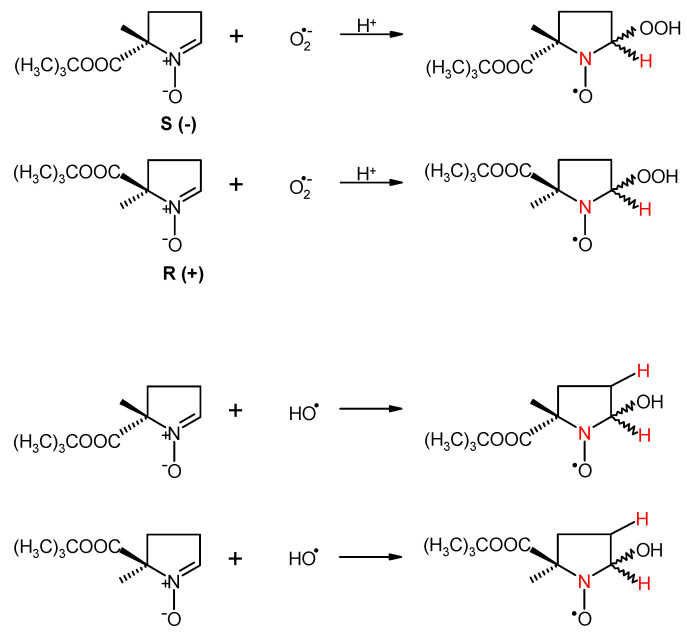
5-*tert*-butoxycarbonyl-5-methyl-1-pyrroline-*N*-oxide (BMPO) spin trap enantiomers and illustration of the diastereoisomeric spin-adducts generation in BMPO reactions with O_2_^•−^ and HO^•^. Nuclei included in the simulation of the experimental EPR spectra of the ^•^BMPO-OOH and ^•^BMPO-OH adducts are marked in red.

The spin-trapping agent BMPO in aqueous solutions represents a racemic mixture of two enantiomers [S (−) and R (+)], and the previous detailed study evidenced that starting with racemate, as well as with the individual enantiomers, the reaction with O_2_^•−^ resulted in the identical EPR spectrum representing two signals of diastereoisomers at the same ratio (*trans* and *cis* with respect to the *tert*-butoxycarbonyl group) (Scheme 1). The EPR spectra of ^•^BMPO-OOH diastereoisomers are characterized with the similar nitrogen splittings and slightly different β-hydrogen splittings [18,19,20]. Analogously, the experimental EPR spectra of ^•^BMPO-OH were interpreted considering the superposition of individual signals of two diastereoisomers with the different hyperfine coupling constants [15,21] (Scheme 1).

Therefore, the aim of our work was to use EPR with BMPO—which in the presence of O_2_^•−^ forms a relatively stable ^•^BMPO-OOH and can serve as a model for organic hydroperoxide—to study the effects of Na_2_S_n_ (*n* = 1, 2, 4) and Na_2_SeO_n_ (*n* = 3, 4) on their own or their mixture Na_2_S_n_/Na_2_SeO_n_. This approach enables the comparison of the relative potential of the investigated compounds to affect the ROOH bond and to eliminate radicals formed during its decomposition. Since 10% DMSO (*v*/*v*) is used in the studied system, the procedure is useful for evaluating antioxidant potency of compounds insoluble in water.

## 2. Materials and Methods

### 2.1. Chemicals

Stock solutions of sodium selenite (Na_2_SeO_3_, 10 or 40 mmol L^−1^, Sigma-Aldrich 214485, Saint Louis, MO, USA) and sodium selenate (Na_2_SeO_4_, 10 mmol L^−1^, Sigma-Aldrich S0882, Saint Louis, MO, USA) were prepared freshly in deionized H_2_O, stored at 23 °C and used within 5 h. Na_2_SeO_3_ dissociates in solution to yield mostly H_2_SeO_3_ at acidic pH, HSeO_3_^−^ at neutral pH and SeO_3_^2−^ at alkaline pH. For simplicity, the terms SeO_3_^2−^ and SeO_4_^2−^ are employed as representative expression to encompass the total mixture of different (de)protonation states. Spin-trapping agent 5-*tert*-butoxycarbonyl-5-methyl-1-pyrroline-*N*-oxide (BMPO, 100 mmol L^−1^, DoJindo B568-10, Munich, Germany) was dissolved in deionized H_2_O, stored at −80 °C and used after thawing. Na_2_S (100 mmol L^−1^) as a source of H_2_S and polysulfides, sodium disulfide (Na_2_S_2_, 10 mmol L^−1^) and sodium tetrasulfide (Na_2_S_4_, 10 mmol L^−1^) (Dojindo SB01, SB02 and SB04, respectively, Munich, Germany) were prepared in argon-bubbled deionized H_2_O, aliquoted, stored at −80 °C and thawed just before use [5]. Na_2_S dissociates in aqueous solution and reacts with H^+^ to yield H_2_S, HS^−^ and a trace of S^2^^−^. We use the term H_2_S to describe the total mixture of H_2_S, HS^−^ and S^2−^ forms. Similarly, Na_2_S_2_ and Na_2_S_4_ dissociate in aqueous solution yielding S_n_^2−^, HS_n_^−^ and traces of H_2_S_n_ (*n* = 2 and 4). For simplicity, we use the terms Na_2_S_2_ and Na_2_S_4_. For EPR samples, buffer consisting of 50 mmol L^−1^ sodium phosphate (pH 7.4, 37 °C) and 100 µmol L^−1^ diethylenetriaminepentaacetic acid (DTPA) was used. Saturated KO_2_/DMSO solution was prepared by the addition of powdered KO_2_ (Sigma-Aldrich 278904, Steinheim, Germany) into the anhydrous DMSO (1.42 mg mL^−1^; 23 ± 1 °C; theoretically 20 mmol L^−1^ KO_2_), vortexed for 2 min, sonicated for 20 s and let for 1 h to settle down the undissolved KO_2_ powder. When an aliquot of KO_2_/DMSO was taken from the bottom part of the stock solution and added into the phosphate buffer, the spectral intensity of ^•^BMPO-OOH was 2- to 4-fold higher than that of the aliquot taken from the upper part [5,13]. For EPR study we used the aliquots of the saturated stock KO_2_/DMSO obtained from the upper part of the stock solution. The intensity of ^•^BMPO-OOH EPR spectra was reproducible when the KO_2_/DMSO stock solution was incubated at 23 ± 1 °C and used within ~4 h.

### 2.2. EPR Study of the BMPO-Adducts

A modified protocol from our previous study was used [13]. First, ^•^BMPO-OOH was prepared by the addition of the saturated KO_2_/DMSO solution (10% *v*/*v* DMSO/final buffer) into the BMPO (30 mmol L^−1^ final concentration, 37 °C) diluted in the phosphate buffer. The BMPO/KO_2_ sample was mixed for 10 s and then the studied compounds, H_2_S_n_, SeO_n_^2−^ or H_2_S_n_/SeO_n_^2−^ mixtures, were added. The sample was mixed again for 5 s and transferred to a standard cavity aqueous EPR flat cell (WG 808-Q, Wilmad-LabGlass, Vineland, NJ, USA). The first EPR spectrum was recorded 100 s after the addition of the compounds. The sets of the individual EPR spectra of the BMPO spin-adducts were recorded as 15 sequential scans, each 42 s, with a total acquisition time of 11 min. Each experiment was repeated at least twice. EPR spectra of the BMPO spin-adducts were measured on a Bruker EMX spectrometer (Rheinstetten, Germany), X-band ~9.4 GHz, 335.15 mT central field, 8 mT scan range, 20 mW microwave power, 0.1 mT modulation amplitude, 42 s sweep time, 20.48 ms time constant and 20.48 ms conversion time at 37 °C. To compare the relative potency of the compounds to decrease overall trapped radical concentration, a second integral of the total EPR spectra intensity of the BMPO-adducts was evaluated. To obtain the ^•^BMPO-OH/^•^BMPO-OOH radical proportion, the simulated spectra ratio was calculated using EasySpin program working on MatLab platform [22].

### 2.3. Plasmid DNA Cleavage

A pDNA cleavage assay with the use of pBR322 plasmid (New England BioLabs, Inc., N3033 L, Ipswich, MA, USA) was performed as reported previously [13]. In this assay, all samples contained 0.2 μg pDNA in the sodium phosphate buffer (25 mmol L^−1^ sodium phosphate, 50 μmol L^−1^ DTPA, pH 7.4). FeCl_2_ (150 µmol L^−1^) was used in control experiments. Powdered KO_2_ was dissolved by 4 mmol L^−1^ BMPO in the buffer containing 10% DMSO (*v*/*v*), vortexed for 10 s and 10 µL of the mixture was added into 10 µL solution of pDNA. The resulting mixtures were incubated for 30 min at 37 °C. After incubation, the reaction mixtures were subjected to 0.6% agarose gel electrophoresis. Integrated densities of all pBR322 forms in each lane were quantified using the Image Studio analysis software (LI-COR Biotechnology, Bad Homburg, Germany) to estimate pDNA-cleavage efficiency. 

## 3. Results

### 3.1. ^•^BMPO-OOH as a Model Hydroperoxide and the Effects of Na_2_S_n_/Na_2_SeO_n_

**Figure 1:**
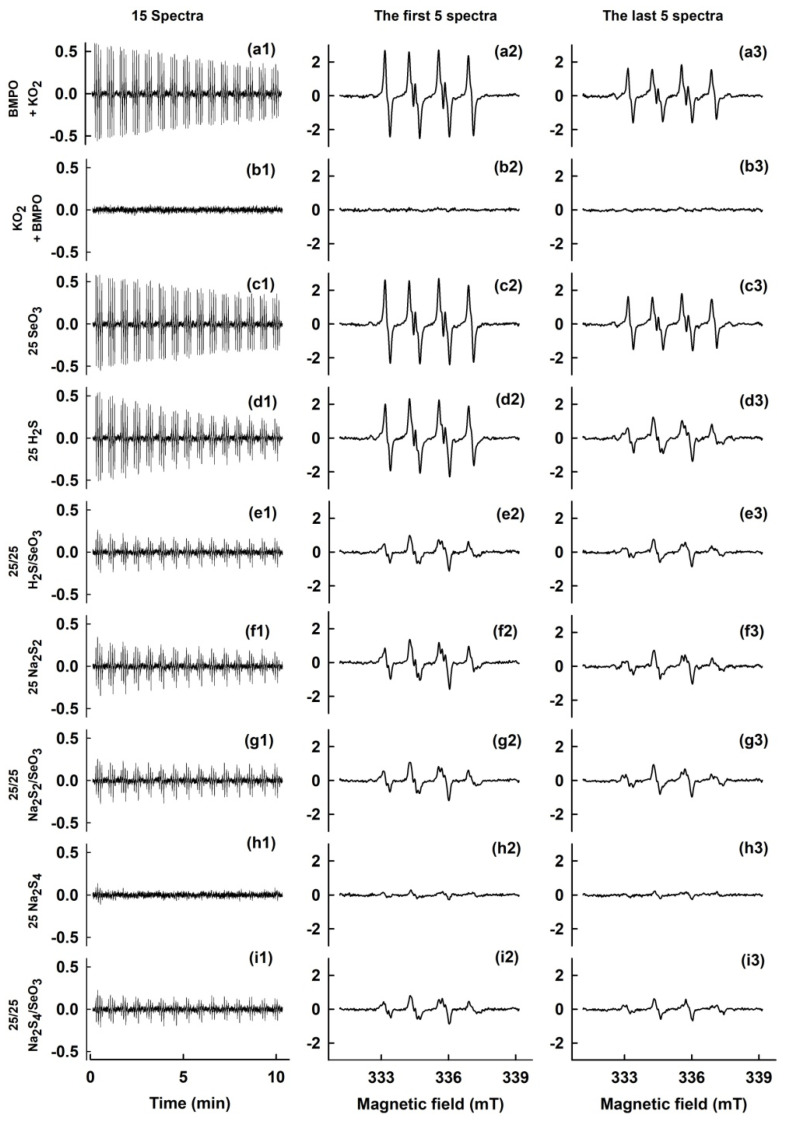
Electron paramagnetic resonance (EPR) spectra of ^•^BMPO-OOH modulated by Na_2_S, Na_2_S_2_, Na_2_S_4_ and their interaction with SeO_3_^2^. (**a1**–**i1**) Collection of 15 EPR spectra arranged back-to-back of the BMPO-adducts, each 42 s, with starting acquisition 100 s after sample preparation; (**a2**–**i2**) first to fifth accumulated spectra; (**a3**–**i3**) last five accumulated spectra; (**a1**–**a3**) representative control EPR spectra from 2–3 measurements of ^•^BMPO-OOH after saturated KO_2_/DMSO solution (final 10% *v*/*v* DMSO) was added to 30 mmol L^−1^ BMPO in the buffer consisting 50 mmol L^−1^ sodium phosphate buffer and 0.1 mmol L^−1^ DTPA (pH 7.4, 37 °C); spectra of ^•^BMPO-OOH after the addition of (**c1**–**c3**) 25 µmol L^−1^ SeO_3_^2−^, (**d1**–**d3**) 25 µmol L^−1^ Na_2_S, (**e1**–**e3**) 25/25 (in µmol L^−1^) Na_2_S/SeO_3_^2−^, (**f1**–**f3**) 25 µmol L^−1^ Na_2_S_2_, (**g1**–**g3**) 25/25 (in µmol L^−1^) Na_2_S_2_/SeO_3_^2−^, (**h1**–**h3**) 25 µmol L^−1^ Na_2_S_4_ and (**i1**–**i3**) 25/25 (in µmol L^−1^) Na_2_S_4_/SeO_3_^2−^. In the case of (**b1**–**b3)** spectra, 30 mmol L^−1^ BMPO was added 10 s after KO_2_/DMSO was mixed with the buffer. The intensities of the (**a1**–**i1**) time-dependent EPR spectra and (**a2**–**i2**, **a3**–**i3**) detailed spectra are comparable; they were measured under identical EPR spectrometer settings.

KO_2_ in DMSO dissociates to K^+^ and relatively stable O_2_^•−^, but its solubility in DMSO is extremely low [23,24]. EPR spectra after the addition of KO_2_ into the BMPO-buffered solution showed signals of two conformers of the ^•^BMPO-OOH adduct, as a result of O_2_^•−^ trapping by BMPO (Figure 1a1–a3). The ^•^BMPO-OOH signal was relatively stable and slowly decreased (t_1/2_ ~23 min) in accordance with [15,16,18]. However, when KO_2_ was added into the buffer first, followed by the addition of BMPO 10 s later no radical was trapped by BMPO, i.e., the EPR spectrum was not observed (Figure 1b1–b3). The results confirmed a short life time of O_2_^•−^ in water solution (t_1/2_ ~1–10 µs). Therefore, we used the interaction of O_2_^•−^ with BMPO, producing the ^•^BMPO-OOH spin-adduct, as a model of organic hydroperoxides to study the effects of Na_2_S_n_/Na_2_SeO_n_. The following sequence of the compounds was used to prepare the sample: KO_2_ was added into BMPO-buffered solution, resulting in the formation of ^•^BMPO-OOH and then the studied compounds were added 10 s later.

Information that can be obtained from the EPR spectra after the interaction of ^•^BMPO-OOH with compounds added into the system is as follows: First, it is an effect of compounds on the total integral EPR intensity of the BMPO-adducts obtained as a second integral of EPR spectra. Second, from the simulation of individual BMPO-adducts of the experimental EPR spectra it may be possible to obtain the changes in their relative concentrations (^•^BMPO-OOH and ^•^BMPO-OH), thereby reflecting the effect of compounds.

The presence of SeO_3_^2−^ (25 µmol L^−1^) did not influence the rate of ^•^BMPO-OOH decomposition (Figure 1c1). From the similar shape of the first five cumulative spectra (Figure 1c2) and the last five accumulated spectra (Figure 1c3), it is assumed that there was no interaction between SeO_3_^2−^ and ^•^BMPO-OOH. The addition of Na_2_S (25 µmol L^−1^; H_2_S) slightly increased the rate of ^•^BMPO-OOH decay (Figure 1d1), as an indication of scavenging/interaction of the BMPO-adducts by/with H_2_S. Since the shape of the last five accumulated spectra (Figure 1d3) was different from the first five spectra (Figure 1d2), it can be concluded that H_2_S affects the ^•^BMPO-OOH spin-adduct. The details on the interaction are described in the next section. The H_2_S/SeO_3_^2−^ (25/25 µmol L^−1^/µmol L^−1^) mixture decreased ^•^BMPO-OOH concentration during 100 s after the sample preparation (before EPR measurement started) and later the BMPO-adducts concentration was approximately constant (Figure 1e1). However, spectra were different (Figure 1e2,e3) from control ^•^BMPO-OOH (Figure 1a2,a3), indicating interaction of H_2_S/SeO_3_^2−^ with ^•^BMPO-OOH. Similar effect was detected when polysulfide Na_2_S_2_ (25 µmol L^−1^) and the Na_2_S_2_/SeO_3_^2−^ (25/25 µmol L^−1^/µmol L^−1^) mixture was used (Figure 1f1–f3,g1–g3). The effects of polysulfide Na_2_S_4_ (25 µmol L^−1^) was the most pronounced, it diminished ^•^BMPO-OOH concentration during 100 s, before EPR measurement started (Figure 1h1–h3). However, its effect to scavenge the BMPO-adducts decreased when the Na_2_S_4_/SeO_3_^2−^ (25/25 µmol L^−1^/µmol L^−1^) mixture was used (Figure 1i1). The spectra (Figure 1i2,i3) revealed pronounced interaction with ^•^BMPO-OOH.

**Figure 2:**
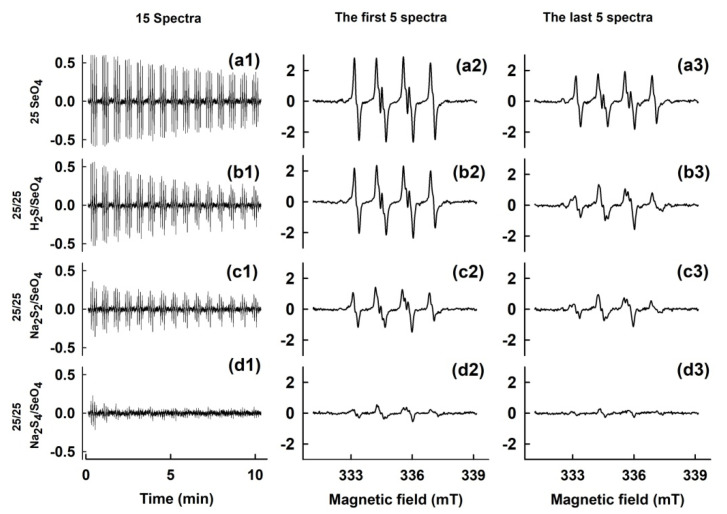
EPR spectra of ^•^BMPO-OOH modulated by the mixture of Na_2_S, Na_2_S_2_ and Na_2_S_4_ with SeO_4_^2−^. Representative EPR spectra from 2–3 measurements of ^•^BMPO-OOH obtained in KO_2_/DMSO solution (final 10% *v*/*v* DMSO) in the presence of 30 mmol L^−1^ BMPO prepared in buffer consisting 50 mmol L^−1^ sodium phosphate buffer and 0.1 mmol L^−1^ DTPA (pH 7.4, 37 °C), after the addition of (**a1**–**a3**) 25 µmol L^−1^ SeO_4_^2−^, (**b1**–**b3**) 25/25 (in µmol L^−1^) Na_2_S/SeO_4_^2−^, (**c1**–**c3**) 25/25 (in µmol L^−1^) Na_2_S_2_/SeO_4_^2−^ and (**d1**–**d3**) 25/25 (in µmol L^−1^) Na_2_S_4_/SeO_4_^2−^. Sets of individual EPR spectra of the BMPO-adducts were recorded as described in legend to Figure 1. The intensities of the (**a1**–**d1**) time-dependent EPR spectra and (**a2**–**d2**, **a3**–**d3**) detailed spectra are comparable, they were measured under identical EPR spectrometer settings.

When SeO_4_^2^^−^ was used instead of SeO_3_^2−^, it did not influence the effects of Na_2_S, Na_2_S_2_ and Na_2_S_4_, indicating no formation of redox active products of the Na_2_S_n_/SeO_4_^2^^−^ interaction (Figure 2).

### 3.2. Simulation of BMPO-Adducts Spectra in the Presence of Na_2_S_n_/Na_2_SeO_3_

**Figure 3:**
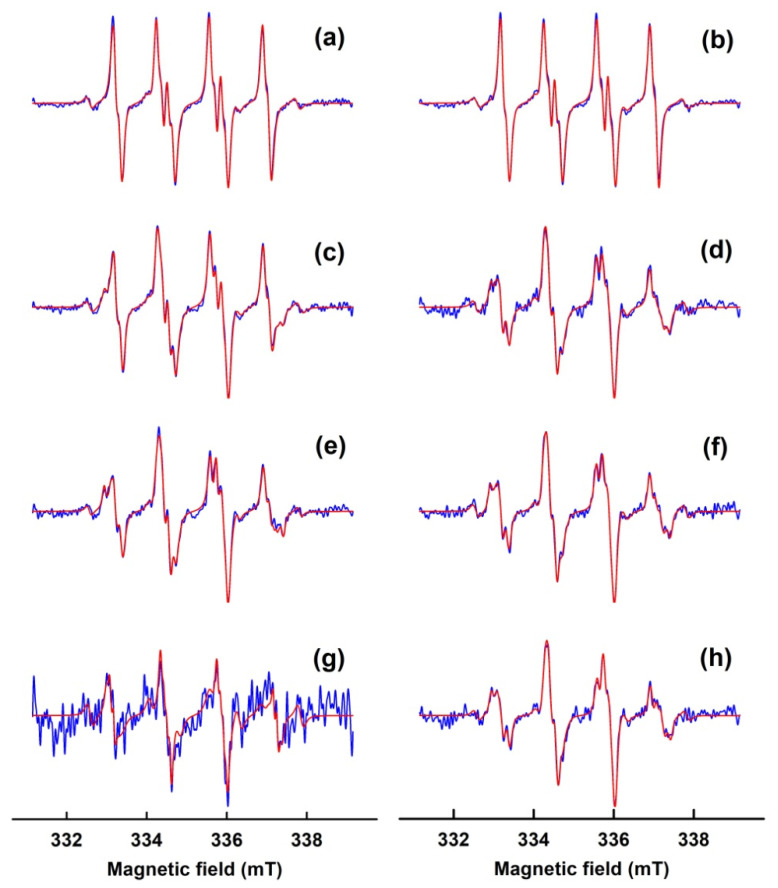
Representative normalized experimental EPR spectra of the BMPO-adducts along with their simulation using the hyperfine coupling constants summarized in Table 1. Experimental spectra of the sixth to tenth accumulated spectra are shown only (blue); simulated spectra are red. (**a**) Control 30 mmol L^−1^ BMPO + KO_2_ and after addition of (**b**) 25 µmol L^−1^ SeO_3_^2−^, (**c**) 25 µmol L^−1^ H_2_S, (**d**) 25/25 (in µmol L^−1^) H_2_S/SeO_3_^2−^, (**e**) 25 µmol L^−1^ Na_2_S_2_, (**f**) 25/25 (in µmol L^−1^) Na_2_S_2_/SeO_3_^2−^, (**g**) 25 µmol L^−1^ Na_2_S_4_ and (**h**) 25/25 (in µmol L^−1^) Na_2_S_4_/SeO_3_^2−^.

The studied compounds changed shapes of EPR spectra of the BMPO-adducts, indicating the superposition of signals corresponding to the generation of individual BMPO-adducts. Therefore, we analyzed all accumulated spectra by simulation. Figure 3 shows representative examples of the simulation of the sixth to tenth accumulated spectra only. The results showed that the best fit was obtained when the hyperfine coupling constants for two conformers of ^•^BMPO-OOH and two of BMPO-hydroxyl radical (^•^BMPO-OH) adducts, along with those of BMPO-adduct with carbon-centered radical (^•^BMPO-CR) were inserted in spin-Hamiltonian calculations. The simulated spectra shown in Figure 3 were calculated using the hyperfine coupling constants elucidated from the experimental spectra (Table 1).

**Figure 4:**
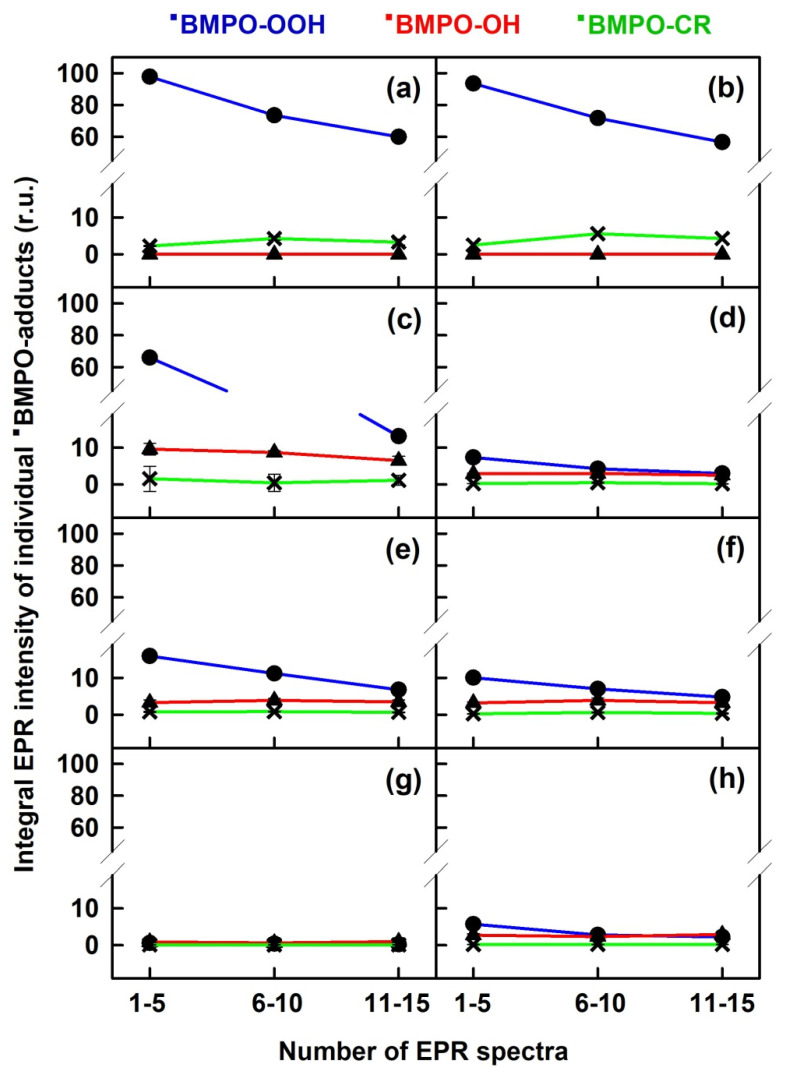
Comparison of integral EPR intensity of individual BMPO-adducts elucidated from the simulation of experimental EPR spectra. The first to fifth accumulated spectra (1–5; 1.7–5.2 min after sample preparation; see Figure 1), the sixth to tenth accumulated spectra (6–10; 5.2–8.7 min after sample preparation) and the eleventh to fifteen accumulated spectra (11–15; 8.7–12.2 min after sample preparation; see Figure 1) Spectral components: ^•^BMPO-OOH (blue), ^•^BMPO-OH (red) and ^•^BMPO-CR (green); *n* = 2–3. (**a**) Control 30 mmol L^−1^ BMPO + KO_2_ and after addition of (**b**) 25 µmol L^−1^ SeO_3_^2−^, (**c**) 25 µmol L^−1^ H_2_S, (**d**) 25/25 (in µmol L^−1^) H_2_S/SeO_3_^2−^, (**e**) 25 µmol L^–1^ Na_2_S_2_, (**f**) 25/25 (in µmol L^–1^) Na_2_S_2_/SeO_3_^2−^, (**g**) 25 µmol L^–1^ Na_2_S_4_ and (**h**) 25/25 (in µmol L^−1^) Na_2_S_4_/SeO_3_^2−^. Data are presented as the means ± SEM.

For simplicity, the relative concentration of two conformers ^•^BMPO-OH(1) and ^•^BMPO-OH(2) were summed up and described as ^•^BMPO-OH. Analogously, ^•^BMPO-OOH(1) and ^•^BMPO-OOH(2) were summed up and described as ^•^BMPO-OOH. The time dependence of such evaluated BMPO-adducts EPR integral intensity is shown in Figure 4. In this figure, the absolute integral of individual BMPO-adducts is depended besides sample composition also on time. For example, the first to fifth accumulated spectra of control integral intensity of ^•^BMPO-OOH spectra (circle; measured 1.7–5.2 min after sample preparation; see Figure 4a tick 1–5) has value 100 (r.u.) and the integrals of ^•^BMPO-OH (triangle) and ^•^BMPO-CR (cross) components of the sample are close to zero. The same control sample is measured 5.2–8.7 min after sample preparation (sixth to tenth accumulated spectra; see Figure 4a tick 6–10) and shows decrease to 73.5 (r.u.) for ^•^BMPO-OOH component, whereas integral of ^•^BMPO-OH is close to zero and ^•^BMPO-CR increased to 4.2.

In controls and samples with SeO_3_^2−^, similar concentration of radicals and a slow decay of the ^•^BMPO-OOH component was seen, indicating no interaction of SeO_3_^2−^ with ^•^BMPO-OOH (Figure 4a,b). The addition of Na_2_S increased the rate of ^•^BMPO-OOH decay (Figure 4c), whereas slightly increased the concentration of ^•^BMPO-OH. The decay was several times pronounced when the H_2_S/SeO_3_^2−^ mixture was used (Figure 4d). Na_2_S_2_ and Na_2_S_4_ alone and their mixture with SeO_3_^2−^ significantly decreased the number of radicals and increased the rate of ^•^BMPO-OOH decay (Figure 4e–h), whereas slightly increased concentration of ^•^BMPO-OH with exception of Na_2_S_4_, where radical concentration was close to zero (Figure 4g). It is noticed that spin adducts concentration in the case of Na_2_S_4_/SeO_3_^2−^ was noticeably higher compared to Na_2_S_4_ alone (Figure 4g vs. Figure 4h). The total integral EPR intensity of all components (Figure 4) revealed that the order of potential to transform/scavenge the BMPO-adducts was Na_2_S_4_ > Na_2_S_2_ > H_2_S (Figure 4c,e,g) and H_2_S/SeO_3_^2−^ > H_2_S (Figure 4c,d), Na_2_S_2_ ~Na_2_S_2_/SeO_3_^2^^−^ (Figure 4e,f), but Na_2_S_4_ > Na_2_S_4_/SeO_3_^2−^ (Figure 4g,h).

**Figure 5:**
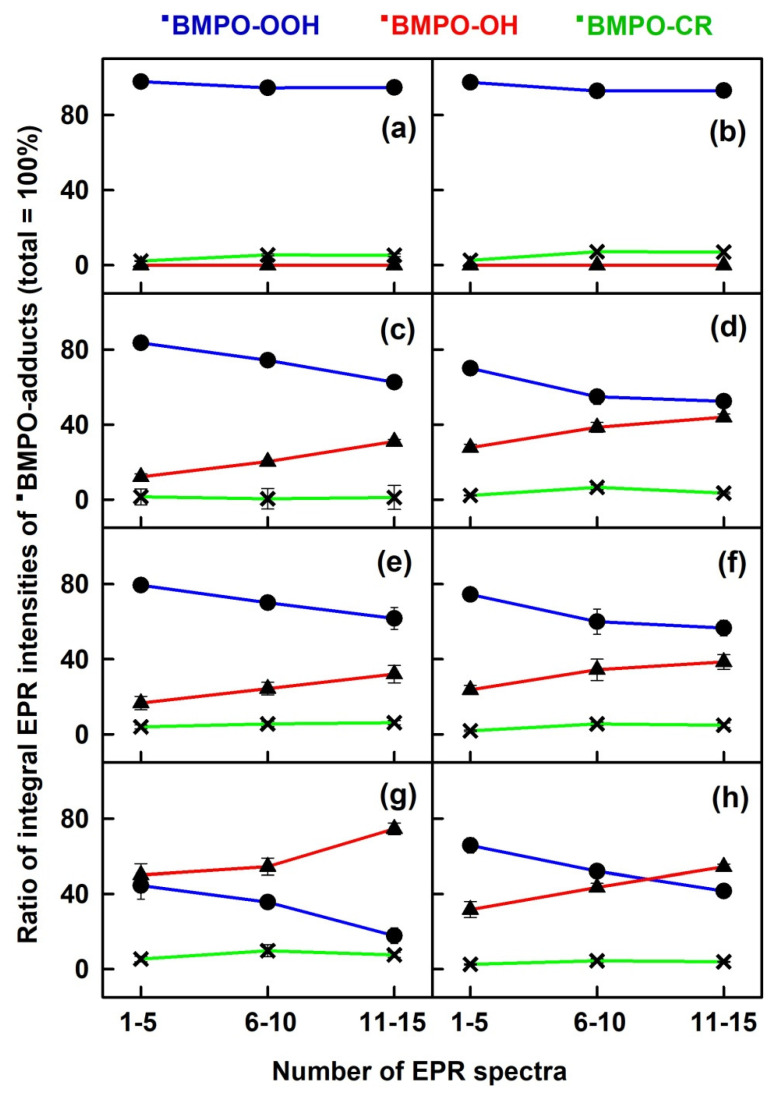
Ratio of normalized integral EPR intensity of individual BMPO-adducts evaluated from experimental spectra simulation. Data calculated from Figure 4. For explanation of (**a–h**) and figure description, see legend to Figure 4.

In order to compare the relative ratio of components of the BMPO-adducts the sum of the radicals at each time was normalized to 100% (Figure 5). In controls and samples with SeO_3_^2−^, the major ^•^BMPO-OOH component was relatively constant over the time with minor ^•^BMPO-CR component, indicating no decomposition of ^•^BMPO-OOH by SeO_3_^2−^ (Figure 5a,b). H_2_S and the H_2_S/SeO_3_^2^^−^ mixture decreased ^•^BMPO-OOH component and increased ^•^BMPO-OH over the time (Figure 5c,d), suggesting time-dependent transformation/decomposition of the model hydroperoxide ^•^BMPO-OOH to ^•^BMPO-OH. Similar qualitative results were observed when Na_2_S_2_ alone or in the combination with SeO_3_^2−^ was used (Figure 5e,f). Na_2_S_4_ alone significantly decreased ^•^BMPO-OOH and increased the ^•^BMPO-OH component (Figure 5g). The Na_2_S_4_/SeO_3_^2^^−^ mixture had similar, but slightly lower effects compared to Na_2_S_4_ (Figure 5h). The order of the compounds potency to cleave ^•^BMPO-OOH, estimated from the time-dependent ^•^BMPO-OH/^•^BMPO-OOH ratio was: Na_2_S_4_ > Na_2_S_4_/SeO_3_^2^^−^ > H_2_S/SeO_3_^2^^−^ > Na_2_S_2_ ~Na_2_S_2_/SeO_3_^2^^−^ ~H_2_S > SeO_3_^2^^−^ ~control.

### 3.3. Cleavage of Plasmid DNA

**Figure 6:**
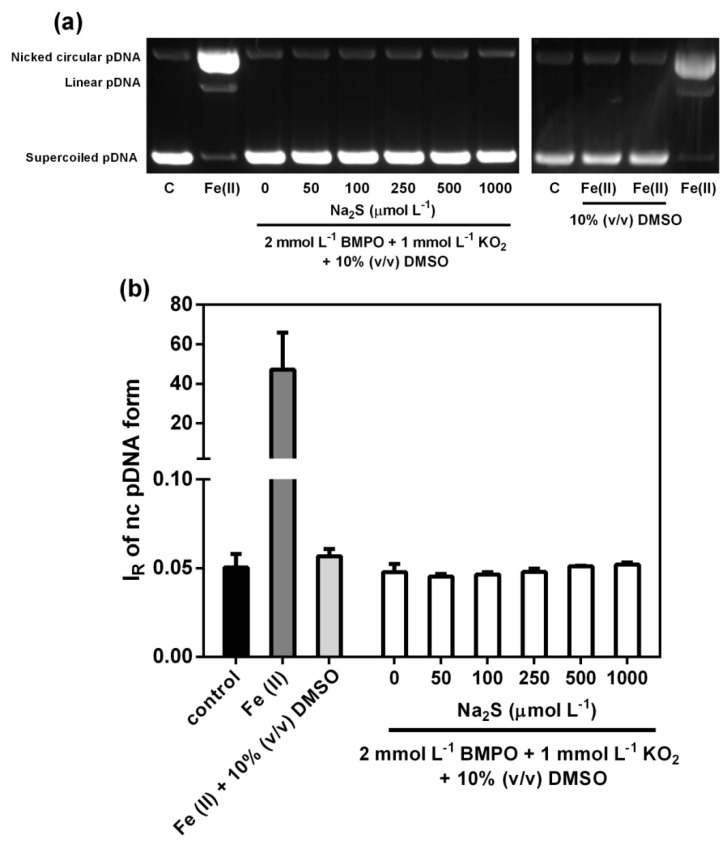
Effect of BMPO/KO_2_ on pDNA cleavage in the absence and presence of Na_2_S. Representative gel (**a**) and column graph (**b**) indicating control (black) and the effects of Fe^2+^ (150 µmol L^−1^ FeCl_2_) in 25 mmol L^−1^ sodium phosphate buffer and 50 µmol L^−1^ DTPA (pH 7.4) without (dark) and with 10% (*v*/*v*) DMSO (gray) and increasing concentrations of Na_2_S on pDNA cleavage in the phosphate buffer containing 10% DMSO (*v*/*v*). The band at the bottom corresponds to the circular supercoiled form of pDNA and the less intense band appearing above in the case of Fe^2+^ (dark column) represents the linear form of pDNA. The top band corresponds to the nicked circular form of pDNA. Values are the means ± SEM, *n* = 3.

Since Na_2_S interacted with ^•^BMPO-OOH (Figure 4 and Figure 5), it was of interest to know whether radical species, which can damage DNA, were formed during the interaction. The procedure for EPR experiments was modified to observe pDNA cleavage. In the control experiment, Fe^2+^ (150 µmol L^−1^) cleaved pDNA. However, when the reaction buffer contained 10% (*v*/*v*) DMSO, Fe^2+^ did not cleave pDNA. Cleaving of pDNA was observed neither in the presence of BMPO/KO_2_ mixture in buffer with 10% (*v*/*v*) DMSO, nor when Na_2_S was added into BMPO/KO_2_/DMSO (Figure 6). From the experiments with Fe^2+^ it is evident that DMSO interfered with the assay. This is supported also by information that DMSO is a scavenger of HO^•^ radicals [13,25]. Therefore, we did not precede with the pDNA experiments.

## 4. Discussion

In our previous study, when KO_2_ as a source of O_2_^•−^ was added into the mixture of H_2_S/BMPO or H_2_S/SeO_3_^2−^/BMPO, the concentration of trapped radicals changed and a superposition of several individual BMPO-adducts was detected [13]. It was not clear which components of the spectra resulted from the compounds/O_2_^•−^ or from the compounds/^•^BMPO-OOH interaction. Therefore, in the present work we used a different approach, which allowed us to study interaction of the compounds with a model of organic hydroperoxide ^•^BMPO-OOH. This approach is based on the short lifetime of O_2_^•−^ in water solution and relatively stable ^•^BMPO-OOH to which the studied compounds were added.

This approach allowed the study of the effects of compounds on decomposition/transformation of the organic hydroperoxide ^•^BMPO-OOH to the ratio of ^•^BMPO-OH/^•^BMPO-OOH, which was detected by EPR. To elaborate this, we performed a detailed simulation of the experimental EPR spectra obtaining the hyperfine coupling constants of the individual BMPO-adducts under these experimental conditions, along with their relative concentrations. From the comparison of the experimental and the simulated EPR spectra (Figure 3), it can be concluded that the BMPO-adducts spectra were very well simulated by the hyperfine coupling constants (Table 1), based on the presence of two conformers, ^•^BMPO-OH(1) and ^•^BMPO-OH(2), ^•^BMPO-OOH(1) and ^•^BMPO-OOH(2) and ^•^BMPO-CR. The hyperfine coupling constants were clearly indicated from the spectra simulations (Table 1) and were comparable to the published BMPO-adducts. The relative intensity of ^•^BMPO-OOH decreased slowly over time and was comparable, but not identical, to the reported values under physiological conditions without DMSO [15,16,18]. It is likely that the ^•^BMPO-CR component resulted from the trapping carbon-centered radical, originating from DMSO. From the time dependence of the integral EPR intensity (Figure 4 and Figure 5) it was possible to evaluate the time-dependent effects of the compounds on the total BMPO-adduct concentration and the ^•^BMPO-OH/^•^BMPO-OOH ratio, which is suggested to be proportional to the organic hydroperoxide ^•^BMPO-OOH transformation/decomposition. The presence of SeO_3_^2−^ potentiated the decrease of integral intensity of spin-adducts induced by Na_2_S, had no significant effect on Na_2_S_2_ potency, but decreased the potency of Na_2_S_4_ (Figure 4). These results indicate that the interaction of SeO_3_^2−^ with Na_2_S_n_, which leads to the formation of redox radical species significantly depends on the number of S atoms.

The order of ability to decrease the total integral intensity of the BMPO-adducts (Figure 4) of H_2_S/SeO_3_^2^^−^ > H_2_S or Na_2_S_4_ > H_2_S was similar to the order when KO_2_ was added to the mixtures of the BMPO/compounds or DEPMPO/compounds [5,13]. This indicates that in the case when KO_2_ was added to the mixtures of spin trap/compounds, the compounds affected mostly ^•^BMPO-OOH or ^•^DEPMPO-OH radicals.

A comparison of the time-dependent ratio of ^•^BMPO-OH/^•^BMPO-OOH (Figure 5) revealed that the increase of the ^•^BMPO-OH spectral component was at the expense of the ^•^BMPO-OOH component, supporting the concept of decomposition/transformation of organic hydroperoxide. Interactions of H_2_S and polysulfides with radical species are complex [4,7] and more studies are needed to explain the most profound potential of Na_2_S_4_ > Na_2_S_2_ or effects of SeO_3_^2^^−^ to decrease the potency of Na_2_S_4_ to cleave ^•^BMPO-OOH.

In conclusion, ^•^BMPO-OOH was demonstrated to be a helpful model of organic hydroperoxide. The presented approach of EPR spectra measurement and analysis of the time-dependent ratio of the ^•^BMPO-OH/^•^BMPO-OOH spin-adducts utilizing the spectra simulation could be useful to study potential of compounds to transform/decompose ^•^BMPO-OOH. Using this approach, the impact of sulfide derivatives (Na_2_S_n_) alone or in the combination with SeO_3_^2^^−^ to transform/decompose ^•^BMPO-OOH was detected and compared. Since 10% DMSO (*v*/*v*) is used in the studied system, the procedure is useful for evaluating antioxidant potency of compounds insoluble in water.

## Figures and Tables

**Table 1:** Hyperfine coupling constants of the BMPO spin-adducts elucidated from the simulations of experimental spectra measured in the buffer solutions containing KO_2_ and 10% DMSO (*v*/*v*). ^•^BMPO-OOH and ^•^BMPO-OH were simulated considering presence of two conformers [15,18,19].

BMPO-Adduct	*a*_N_, mT	*a*_H_^β^, mT	*a*_H_^γ^, mT
^•^BMPO-OH(1)	1.423 ± 0.011	1.541 ± 0.014	0.078 ± 0.011
^•^BMPO-OH(2)	1.365 ± 0.038	1.248 ± 0.036	0.073 ± 0.015
^•^BMPO-OOH(1)	1.339 ± 0.002	1.186 ± 0.007	–
^•^BMPO-OOH(2)	1.334 ± 0.003	0.958 ± 0.007	–
^•^BMPO-CR	1.528	2.221	–
